# Quantification of anisotropy and fiber orientation in human brain histological sections

**DOI:** 10.3389/fnint.2013.00003

**Published:** 2013-02-01

**Authors:** Matthew D. Budde, Jacopo Annese

**Affiliations:** ^1^Department of Neurosurgery, Medical College of WisconsinMilwaukee, WI, USA; ^2^The Brain ObservatorySan Diego, CA, USA; ^3^Department of Radiology, University of California San DiegoSan Diego, CA, USA

**Keywords:** anisotropy, diffusion tensor imaging, structure tensor, crossing fibers, histology, fiber orientation

## Abstract

Diffusion weighted imaging (DWI) has provided unparalleled insight into the microscopic structure and organization of the central nervous system. Diffusion tensor imaging (DTI) and other models of the diffusion MRI signal extract microstructural properties of tissues with relevance to the normal and injured brain. Despite the prevalence of such techniques and applications, accurate and large-scale validation has proven difficult, particularly in the human brain. In this report, human brain sections obtained from a digital public brain bank were employed to quantify anisotropy and fiber orientation using structure tensor analysis. The derived maps depict the intricate complexity of white matter fibers at a resolution not attainable with current DWI experiments. Moreover, the effects of multiple fiber bundles (i.e., crossing fibers) and intravoxel fiber dispersion were demonstrated. Examination of the cortex and hippocampal regions validated-specific features of previous *in vivo* and *ex vivo* DTI studies of the human brain. Despite the limitation to two dimensions, the resulting images provide a unique depiction of white matter organization at resolutions currently unattainable with DWI. The method of analysis may be used to validate tissue properties derived from DTI and alternative models of the diffusion signal.

## Introduction

The brain is a network with multiple levels of connectivity. The connections of each neuron determine its functional properties, and the functionality of the brain as a whole is determined by both these local and large-scale connections. Since anatomical and functional connectivity are intrinsically linked, a major goal of neuroscience has been to construct comprehensive maps of brain connectivity, or “connectomes” (Sporns et al., 2005; Sporns, 2011). Given the many levels of connectivity, from the microscopic to the macroscopic, the tools used to create such maps vary (Leergaard et al., 2012). No single modality is currently capable of reconstructing all connections in the human brain (Kleinfeld et al., 2011). Thus, a number of methods are needed to bridge the gap from the connectivity of individual neurons to those that measure large-scale patterns of anatomical connectivity.

Diffusion weighted imaging (DWI) has become an indispensible tool to investigate the large-scale anatomical connectivity in the brain. As a completely non-invasive modality, DWI has enabled unprecedented insights into brain microstructure and connectivity. Technological advances in the acquisition, analysis, and modeling of DWI data have propelled investigations of the normal brain architecture and the consequences of disease and injury. Despite these achievements, cross-validation of diffusion imaging and microscopy has been somewhat limited. Since DWI measures the mobility of water molecules, it is an indirect measure of structure. Inferences and mathematical models form the basis to derive structural information from the DWI signal. Diffusion tensor imaging (DTI) employs a single tensor model to obtain measures of primary fiber orientation and anisotropy. However, the DTI model has well-known limitations in brain regions that are composed of more than a single coherent fiber bundle, which occurs at high proportions in the human brain (Jeurissen et al., 2012). To remedy these limitations, other models of the DWI signal have been developed to resolve complex fiber orientations and more accurately map intersecting fiber pathways (Wedeen et al., 2012). Additionally, other interesting properties of brain tissue that may be relevant and important in both health and disease have been extracted from models of the DWI signal, such as neurite densities (Jespersen et al., 2010; Zhang et al., 2012), fiber dispersion (Sotiropoulos et al., 2012), and axonal diameters (Barazany et al., 2009; Zhang et al., 2011). However, robust methods of validating these features using other modalities have been limited.

Microscopy remains the standard for definitive examination of tissue microstructure. Post-mortem assessments of tissue organization using optical or electron microscopy are essential to directly visualize cellular and subcellular structure. Often, these methods are limited to small tissue regions and rely on selective sampling of brain tissues. In recent years, advances have been made in large-scale microscopic imaging of whole-brain sections (Denk and Horstmann, 2004; Dodt et al., 2007), including human brains (Annese, 2012). The vast increases in both resolution and coverage have necessitated quantitative methods to extract pertinent information and reduce the data to a manageable format for dissemination. With these advances, microscopy techniques are converging with DWI to enable a comprehensive map of brain microstructural anatomy and connectivity. This convergence will enable cross-validation and will provide insight into the enormous structural complexity of the brain. These insights will advance the understanding of nervous system disease and injury and therefore has considerable importance.

In this report, a method to quantify microscopic tissue properties was applied to human brain sections. Specifically, high-resolution images of whole-human brain sections obtained from a publically available collection underwent structure tensor analysis (Budde and Frank, 2012) to extract microscopic features of anisotropy and orientation, since these are the most common parameters derived from DTI. The work highlights specific features of the brain that are commonly investigated in DTI and other DWI studies, including the effect of crossing fibers and fiber dispersion. Insights to specific anatomical regions, including the cortex and hippocampal structures (Augustinack et al., 2010; Yassa et al., 2010) is also presented. The objective was to introduce the technique as a complement to previous and future DWI studies of the human brain, since accurate validation has been notoriously lacking (Hagmann et al., 2010). The results and methodology outlined here will be useful in validating DTI and advanced models of the diffusion MRI signal and reveal features of the microstructure unattainable with resolution of current DWI examinations.

## Materials and methods

### Data analysis

Human brain sections stained were obtained from a public histological resource for the human brain that is available on The Brain Observatory's web site (2012). Frozen sections were cut at an interval of 70 μm, mounted on glass slides, and stained using a modified silver stain for myelin (Gallyas, 1979). Staining was performed in large-batches, so all materials analyzed in the context of this communication underwent the same protocol. The penetration of the silver salts during impregnation and homogeneous development of the salts into visible colloidal silver was tested empirically on the finished slides using a high-magnification, and high numerical aperture oil immersion objective (Olympus PlanApo N 60×, NA: 1.42). The digitization of sections was performed using a custom-engineered, large-format, slide-scanning microscope using a 20× objective. Images were acquired at a resolution of 0.37 μm^2^ per pixel. The analysis described below used images from 15 whole-brain coronal sections subsampled at a working resolution of 2.96 μm^2^ per pixel. After fixation in 4% formaldehyde phosphate-buffered solution at 4°C, the brain specimen was cryoprotected in increasing concentration of sucrose and embedded using stereotaxic landmarks (Talairach and Tournoux, 1988). Accordingly, sections were obtained approximately perpendicular to a line intersecting the anterior and posterior commissures (Annese, 2012). Further details of tissue processing, including sectioning, staining, and digitization are provided in Bartsch et al. (2012).

### Data analysis

**Figure 1:**
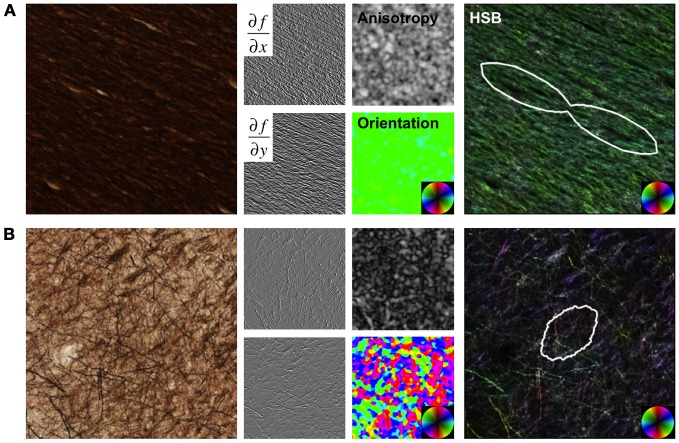
**Structure Tensor Analysis.** Images from a region of high anisotropy in the corpus callosum **(A)** and a region with low anisotropy from the cortical gray matter **(B)** are shown as examples. Original images were convolved with Gaussian derivative filters, and maps of anisotropy and orientation were derived from the structure tensor matrix at each pixel in the image. Hue-saturation-brightness (HSB) images visually display the orientation, anisotropy, and intensity (inverted for these sections), respectively, in a single image. The angular histogram of orientation over the image are quantified and displayed as fiber orientation distributions (FODs).

Structure tensor analysis of histological sections was previously described for the rat brain (Budde and Frank, 2012) and was derived from original methods proposed by Bigun and Granlund (1987). All procedures were implemented as custom routines in Matlab. Briefly, images were convolved with a discrete approximation of 2D Gaussian derivative filters (Figure 1) separately along $x,G_{x}=-xe^{-\frac{x^{2}+y^{2}}{2\sigma^{2}}}$ and $y,G_{y}=-ye^{-\frac{x^{2}+y^{2}}{2\sigma^{2}}}$ to obtain the image partial derivatives, *f*_x_ and *f*_y_, respectively, where σ is the width of the Gaussian window in pixels that defines the inner, or local scale. The structure tensor, *J*, of an image is a matrix representation of the image partial derivatives for each pixel

$$J=\left[\begin{array}{cc} f_{x}^{2} & f_{x}f_{y}\\ f_{x}f_{y} & f_{y}^{2} \end{array}\right]\;\times \;w(x,y).$$

where *w* is a Gaussian convolution kernel of width σ that defines the outer, or integration scale. The primary orientation, corresponding to the direction of the largest eigenvector of the tensor, was obtained by

$$\theta =\frac{1}{2}\text{arctan}\left(2\frac{f_{x}f_{y}}{f_{y}^{2}-f_{x}^{2}}\right)$$

and anisotropy index was obtained by

$$\text{AI}=\frac{\lambda_{\text{1}}-\lambda_{\text{2}}}{\lambda_{1}+\lambda_{2}}$$

where λ_1_ and λ_2_ are the largest and smallest eigenvalues of *J*, respectively. For visualization of images in their native resolution, the values of orientation, anisotropy, and staining intensity were reflected as hue, saturation, and brightness (HSB) images, respectively. Alternatively, downsampling was employed to mimic the resolutions achievable by MRI by summing *f*^2^_*x*_, *f*_*y*_2, and *f*_*xy*_ from regularly spaced square subregions of a predetermined size (i.e., “voxels”) to derive *J* as shown above by omitting the outer scale. In this representation, the primary orientation and anisotropy were displayed as hue and brightness, respectively that mimic directionally encoded color (DEC) images derived from DTI. Additionally, fiber orientation distributions (FODs) were obtained for each voxel for visualization and quantification of the complete in-plane angular profile. The FODs were computed as histograms of pixel orientations, θ, within each voxel using 64 equally spaced bins. To quantify fiber dispersion, FODs were fit to a single Gaussian function $f(\theta )=\frac{1}{\sigma \sqrt{2\pi }}e^{-{\left(\theta -\mu \right)}^{2}/(2\sigma^{2})}$ with a mean orientation of μ and standard deviation of σ using a non-linear least squares routine in the Matlab Curve Fitting toolbox. The standard deviation was displayed as a scalar map to depict regional variations in intravoxel dispersion within the corpus callosum (CC).

## Results

### White matter microstructure

**Figure 2:**
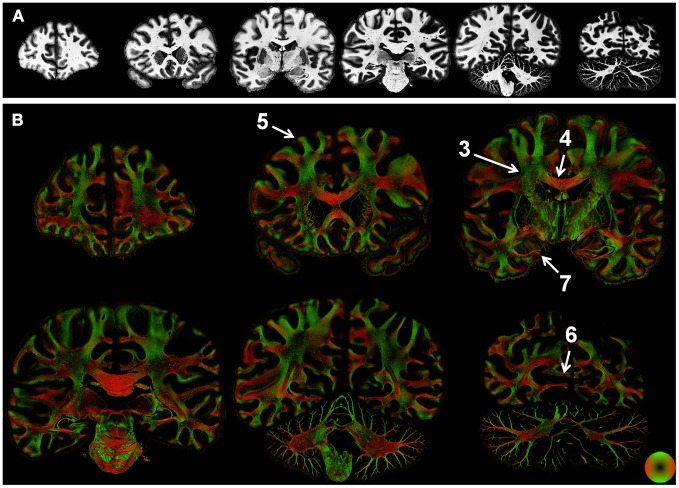
**Whole-section maps of fiber microstructure.** Images of the (inverted) staining intensity **(A)** and directionally encoded color maps (DEC; **B**) were reconstructed at a resolution of 68 μm^2^. Staining intensity was nearly uniform in the white matter, but the DEC images highlight the intricate microstructure of white matter tracts. Numbers indicate the regions shown in greater detail in subsequent figures.

Microscopic tissue orientation and anisotropy was derived using structure tensor analysis applied to images of human brain sections acquired at sub-micron resolution. Myelin staining intensity was largely uniform across the cerebral white matter (Figure 2A) and did not distinguish individual tracts. In contrast, structure tensor analysis revealed individual tracts and the microscopic heterogeneity of the white matter. The derived orientation and anisotropy reflects the microscopic composition of white matter tracts (Figure 2B). Specifically, the CC is the most prominent white matter structure in the brain. As expected, the anisotropy of the CC was greater than the other white matter tracts in the brain. Along with regional variations in orientation indicated by hue, local variations in anisotropy were evident throughout the white matter. Since anisotropy in the current study reflects fibers oriented within the section, low anisotropy could be caused by through-plane fibers. Alternatively, regions that contain multiple fiber populations within a single voxel would also have an anisotropy less than that of coherent fiber bundles. These two effects could not be disambiguated by the current approach. Nonetheless, many large-tracts such as the optic radiations and pathways in the pons were readily visualized in the color-coded images.

**Figure 3:**
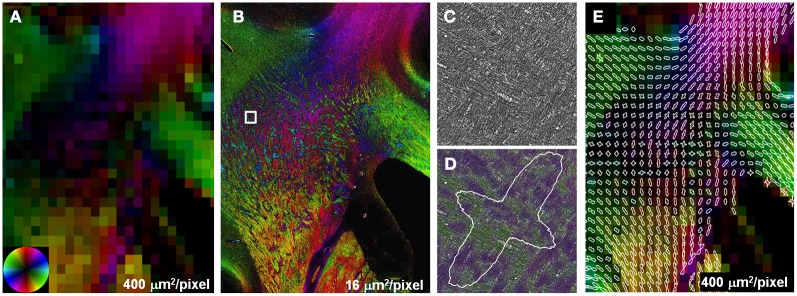
**Partial volume effects and crossing fibers.** Anisotropy in regions containing multiple intersecting fiber tracts was lower compared to single, coherent fiber pathways **(A)**. Reconstructions at higher resolution **(B)** improve the visualization of small, interdigitated fiber bundles in the region containing crossing fibers **(B)**. Compared to the unmodified stained sections **(C)**, crossing fibers were more apparent in the same images color-coded with orientation **(D)**. The two peaks in the FOD highlight the presence of two discrete fiber populations within this subregion of the centrum semi-ovale **(E)**.

The effect of crossing fibers was further demonstrated in a region within the centrum semi-ovale, where fibers of the longitudinal fasciculus and corticospinal tract intersect with transcallosal fibers (Wedeen et al., 2012). DEC images were reconstructed with square pixels of 400 μm^2^ (Figure 3A), substantially smaller than those normally used for whole-brain studies at field strengths of 3T and below. Anisotropy was lower in the region of multiple fiber intersections than adjacent fiber bundles with a single, coherent orientation such as the CC. Reconstructions at high-resolution, 16 μm^2^, resulted in greater anisotropy than the lower resolution, which can be attributed to the decreased effect of partial volumes. The interdigitated fiber pathways were also more readily visualized in the high-resolution reconstructions (Figure 3B). Compared to the unmodified images at their original resolution, 0.37 μm^2^, the same images color-coded with the local fiber orientation clearly revealed that the intersection of major fiber pathways is composed of small fiber bundles. Likewise, the two separable peaks in the FOD depicted the existence of two orthogonal (in-plane) fiber populations.

**Figure 4:**
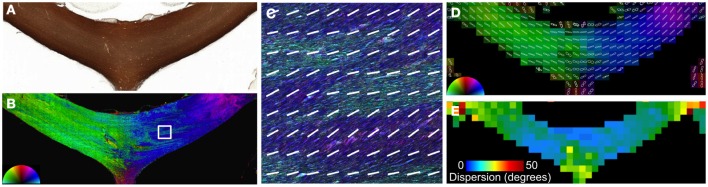
**Angular dispersion of the corpus callosum.** In the body of the CC **(A)**, the microscopic heterogeneity is apparent on images color coded by orientation reconstructed at high resolution **(B** and **C)**. The FODs **(D)** were fit to a Gaussian function to quantify the microscopic orientation dispersion **(E)**. The medial and lateral aspects of the CC had greater dispersion in this section.

In addition to crossing fibers, bending and dispersion of fibers within a single voxel can affect the estimated fiber orientation in diffusion MRI data. The CC is the most prominent structure in the human brain and is considered to consist of a single, coherent fiber pathway. Although the primary direction of fibers in the CC is largely consistent and follows the macroscopic curvature (Figure 4), there are regional variations in orientation that are visualized using structure tensor analysis of high-resolution microscopy data. Smaller fiber bundles that exhibit slightly differences in orientation are likely related to the foliated structure of the CC (Wiggins et al., 2010). The microscopic dispersion was quantified by fitting the FODs from each reconstructed voxel to a Gaussian function (Figure 4), where the standard deviation of the Gaussian fit curve reflects the intravoxel dispersion of fibers. The increased dispersion within the medial and lateral regions of the CC was evident as a broadening of the FODs and the corresponding map of dispersion. Across the whole CC in this section, the mean intravoxel dispersion was 14.6 ± 3.6°.

### Cortical microstructure

**Figure 5:**
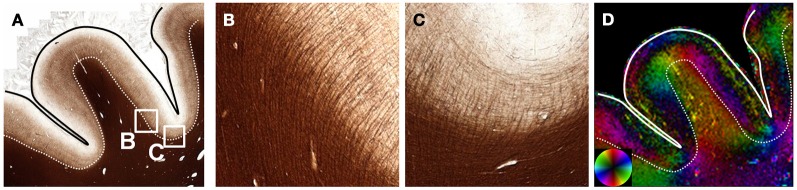
**Cortical Structure.** The myelinated fibers of the cortex **(A)** are predominantly oriented perpendicular to the cortical surface. Within the gyrus, white matter fibers are oriented along the main axis of the gyrus. However, **(B)** fibers entering the cortex from the white matter turn at nearly right angles as evidenced by the abrupt changes in orientation **(D)** along the white-gray matter interface (dotted line). In cortex at the deepest portion of the sulci, the cortex has a mixture of radial and tangential fibers **(C)**. The white matter fibers just below the deepest part of the sulci are oriented tangentially, consistent with the U-shaped association fibers.

The human cerebral cortex has a highly intricate and complex architecture that has been investigated with DWI. High-resolution *in vivo* or *ex vivo* DTI has consistently identified the cortex as anisotropic with its predominant orientation perpendicular to the cortical surface (Heidemann et al., 2012). In an example from the frontal cortex (Figure 5), structure tensor analysis confirms the radial organization in the cortex having high anisotropy. While the white matter fibers within the gyri were perpendicular to the gyral surface throughout, white matter fibers had sharp bends into the cortex (Figure 5B) along the sulcal surface as shown previously with DWI (Takahashi et al., 2011). In the depths of the sulcus, the cortical myeloarchitecture consisted of fibers arranged predominantly parallel to the white- and gray matter interface (Figure 5D). The white matter fibers just superficial to the interface were largely composed of U-shaped association fibers that follow the trajectory of the sulcal depths (Catani et al., 2012).

**Figure 6:**
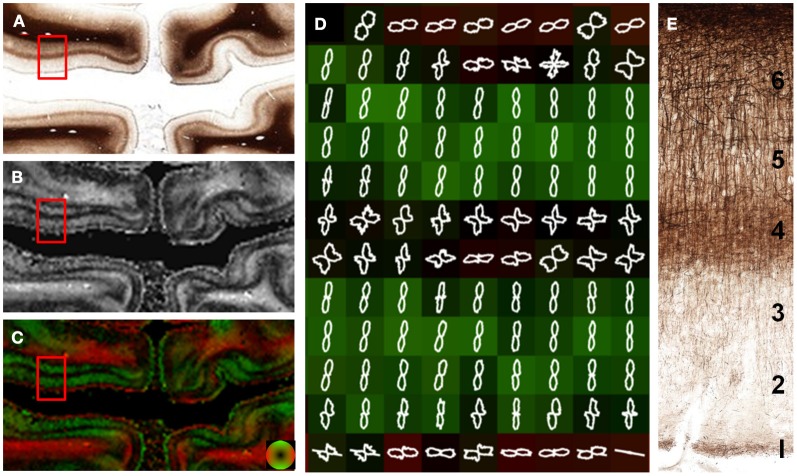
**Laminar-specific anisotropy in striate cortex.** A section from the primary visual cortex **(A)** reveals the highly anisotropic myeloarchitecture of area V1 with a clear laminar subdivision. Layer 1 is predominantly tangential (parallel) to the cortical surface as evidenced by the high anisotropy **(B)** and orientation (red; **C** and **D**). In contrast, layers 2, 3, and 5 have a radial orientation (green; **C** and **D**) with high anisotropy **(B)**, despite their different myelin content. Layers 4 and 6 are composed of both radial and tangential fibers as evidenced by the low anisotropy **(B)** in these regions and the orthogonal peaks in the in the overlaid FODs **(D)**. The microscopic organization of the myelin-stained cortex is shown in **(E)**.

The visual, or striate cortex (Figure 6), displays an architecture different than most other cortical regions. Again, the gray matter was predominantly anisotropic with a radial organization, but different cortical layers were exceptions. Fibers in layer 1 were arranged parallel to the cortical surface. Layers 4 and 6 consisted of fibers arranged both parallel and perpendicular to the cortical surface. This was evident as decreased anisotropy as well as the two opposing peaks in the FOD (Figure 6D), and may be the cause of decreased anisotropy measured with DTI at the gray and white matter interface (Guilfoyle et al., 2003). Although layers 2–3 and 5 have different myelin contents, the anisotropy and orientation information derived from these two layers was similar. The lamina-specific orientations are consistent with *ex vivo* DTI findings by Leuze et al. (2012).

### Perforant pathway

**Figure 7:**
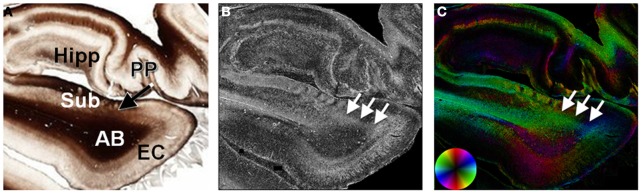
**Histological evidence of the perforant pathway.** The perforant pathway is a set of fibers projecting from the entorhinal cortex through the angular bundle and subiculum to the hippocampal formation **(A)**. In the myelin-stained section, the perforant pathway is seen as it penetrates the subiculum, but is not discriminated from the angular bundle. In the maps of anisotropy **(B)** and orientation **(C)**, the perforant pathway is evident as a region of high anisotropy (arrows) projecting from the entorhinal cortex through the subiculum. AB, angular bundle; EC, entorhinal cortex; Hipp, hippocampal formation; PP, perforant pathway; Sub, subiculum.

The identification of small structures using *in vivo* or *ex vivo* diffusion MRI could have implications as biomarkers of disease activity. The perforant pathway (PP) has been identified in the human brain *in vivo* (Yassa et al., 2010) and *ex vivo* (Augustinack et al., 2010) and has implications to aging and dementia. The PP is a fiber bundle within the medial temporal lobe that connects the hippocampus to the entorhinal cortex. Structure tensor analysis was applied to a human brain section obtained at the level of the hippocampus. Although the PP was evident as a diffuse, myelinated structure within the unmyelinated subiculum (Figure 7), its projection through the myelinated angular bundle was obscured on the myelin-stained section (Figure 7A). However, the map of anisotropy depicted the PP as a diffuse region of high anisotropy (Figure 7B). The orientation of the PP differentiated it from the angular bundle and was consistent with its projection from the entorhinal cortex to the hippocampus (Figure 7C).

## Discussion

Diffusion MRI is unique in its ability to examine the microstructure and large-scale connectivity of the brain non-invasively. However, since measurements of water mobility are an indirect measure of structure, validation with other techniques that directly assess structure are important for robust interpretations of the DWI signal and parameters derived from it. In this report, we derived anisotropy and fiber orientations directly from stained histological sections of the human brain. The results demonstrate that structure tensor analysis is a robust method to extract microstructural features of the brain. The results directly demonstrate the limitations of DTI in regions of complex fiber crossings, and the effect of resolution of the measured anisotropy. The limitations of DTI are analyzed thoroughly in a recent paper by Jones et al. (2012). Furthermore, the method reveals the microstructural properties of anatomical regions that have been previously studied with DWI. For example, in addition to showing the anisotropy and radial organization of the cerebral cortex, the crossing fibers in specific cortical layers have implications to DTI and multi-fiber models. Visualization of other structures of interest, such as the PP, was facilitated by quantitative measures of anisotropy and direction, as these myelinated fibers are difficult to appreciate in myelin-stained sections. The method and results described herein may prove useful in relating microscopy-based measures of structure and connectivity with *in vivo* and *ex vivo* DWI (McNab et al., 2009; Miller et al., 2011, 2012) of the human brain.

The current work has several limitations. Among them, the lack of a third dimension is the most important consideration that limits the potential of the work and has been previously discussed (Budde and Frank, 2012). While the crossing fiber problem could be directly visualized at high-resolution (Figure 3), the lack of three-dimensional information precluded reconstruction of fiber tracts and connectivity maps that reveal unique properties of brain organization (Wedeen et al., 2012). Microscopy techniques to image the brain in three-dimensions have been demonstrated for small tissue samples, such as rodent brains (Dodt et al., 2007; Keller and Dodt, 2011; Mikula et al., 2012). Large-scale application to the human brain is challenging, but not impossible (Bartsch et al., 2012). DTI and other models of characterizing brain structure with diffusion MRI have shown tremendous potential to study the normal and diseased microscopic anatomy. However, validation remains an important, but often overlooked, aspect of studies. As we have shown, the analysis of stained histological sections provides valuable corroborating information that complements diffusion MRI findings from previous *in vivo* and *ex vivo* experiments. A second limitation of the current study is the use of myelin-stained sections. In the white matter, the myeloarchitecture accurately reflects the microstructure as a whole. This feature is exploited in polarized light microscopy to quantify the microstructural organization of tissue in thick sections, including through-plane fibers (Axer et al., 2011a,b). However, in the cortex and subcortical structures, the relatively greater proportions of other cells and subcellular elements, including neuronal somas, dendrites, and glial cells, all contribute to the measured diffusion signal. Since biological membranes are the primary impediments to water mobility in the brain, the use of lipophilic dyes (Budde and Frank, 2012) or serial electron microscopy (Denk and Horstmann, 2004) may also prove essential to validate the tissue properties being probed by advanced diffusion models.

The current work both supports observations highlighted in previous diffusion MRI studies and adds greater insight into the microanatomy of the human brain. In the visual cortex, Leuze et al. recently demonstrated the laminar-specific orientation of the human visual cortex and high-resolution using *ex vivo* DTI and PLM (Leuze et al., 2012). The current work supports the finding that the cortex is predominantly arranged radial to the cortical surface with notable exceptions. Brodman layer 1 is oriented tangentially to the cortical surface and has high anisotropy. In contrast, layer 4 exhibits both radial and tangential fibers and therefore has a much lower anisotropy. At the interface of the white and gray matter, low anisotropy is observed in this study and previous diffusion MRI studies (Miller et al., 2011). The radial and tangential organization of fibers in layer 6 is demonstrated. However, within the visual sulcus, the cortical fibers are orientated radial to the cortex, whereas the underlying white matter fibers are largely tangential to the surface. In the majority of brain regions, fibers that project to the cortex within the sulci have sharp bends at the white-gray matter interface (Takahashi et al., 2011), while fibers projecting to the gyri maintain their straight trajectories into the gyri. These features are highlighted in Figure 5 and may underlie the diffusion MRI findings that gyri and sulci have differing cortical fiber orientations (McNab et al., 2012) and axonal termination densities (Nie et al., 2012).

The quantitative findings of intravoxel dispersion measured in the current study (14.6°) agree with previous studies of the rat brain (14.4; $\text{FWHM}=2\sqrt{2ln2}\cdot \sigma$) (Leergaard et al., 2010) or the owl monkey (12°) (Choe et al., 2012). These histological-derived measures of dispersion were generally consistent with estimates of the same parameter obtained with diffusion MRI using a model that incorporated intravoxel bending or fanning (Sotiropoulos et al., 2012) although variations in the regional pattern within the CC may be different. Since the CC is routinely considered to be composed of a single, coherent fiber population, it is often employed to derive a deconvolution kernel for multiple-fiber models (Tournier et al., 2004). Our high-resolution orientation maps of the CC (Figure 4C) highlight the microscopic variation within the CC that could potentially influence the accuracy of multiple-fiber models (Parker et al., 2013). The inclusion of multiple subjects and additional sections, including those obtained from different orientations, would provide a more comprehensive analysis of the effects of intravoxel dispersion. Importantly, since fractional anisotropy measured with DTI has a direct relationship with fiber density (Choe et al., 2012) but an inverse relationship with fiber dispersion (Zhang et al., 2012), these features might also be important physical phenomenon underlying FA variability in different regions of the brain and across different groups.

### Conflict of interest statement

The authors declare that the research was conducted in the absence of any commercial or financial relationships that could be construed as a potential conflict of interest.
